# Health-related quality of life in children with PFAPA syndrome

**DOI:** 10.1186/s13023-018-0878-3

**Published:** 2018-08-09

**Authors:** Claire Grimwood, Isabelle Kone-Paut, Maryam Piram, Linda Rossi-Semerano, Véronique Hentgen

**Affiliations:** 1 Department of Pediatrics, Ambroise Paré hospital, University of Versailles SQY, 9 avenue Charles de Gaulle, 92100 Boulogne Billancourt, France; 2 CEREMAIA, Pediatric rheumatology, Bicêtre hospital, APHP, university of Paris Sud, Le Kremlin Bicêtre, France; 3 CEREMAIA, departement of pediatrics, Versailles hospital, Versailles Le Chenay, France

**Keywords:** Hereditary auto inflammatory diseases, Quality of life, PFAPA, Familial Mediterranean fever, Child, Preschool child, Fatigue

## Abstract

**Background:**

Conventionally, PFAPA syndrome is considered as a benign disease compared to other recurrent fevers because it completely passes before adulthood. However, in our clinical practice, fever episodes have a huge impact on daily activities.

**Methods:**

Observational cohort study using the Pediatric Quality of Life Inventory (PedsQL™ 4.0) Generic Core and Fatigue Scales. PedsQL™ uses a modular approach to measure the HRQOL in children with acute and chronic health conditions. We used pediatric FMF patients as the control group.

**Results:**

We included 33 children with PFAPA and compared them to 27 FMF patients matched for age: preschool-age children (2 to 7 years) and school-age children and youths (8 to18 years). PedsQL™ self-reported scores of children with PFAPA were systematically lower than those of FMF peers for general quality of life and physical and psychosocial functioning (significant only in the preschool-age group). PedsQL™ self-reported fatigue scores of children with PFAPA were significantly lower than those of FMF peers for both preschoolers and school-age children and youths. Parent proxy-reports were not significantly different, even though scores were systematically lower for the parents of PFAPA children.

**Conclusion:**

Our study demonstrates, for the first time, that the wellbeing of PFAPA children is poor, with a major impact on psychosocial functioning and increased fatigue. The quality of life of PFAPA children appears to be even lower than that of FMF patients, for whom a lower than normal HRQOL has already been demonstrated.

## Background

Periodic Fever, Aphtous stomatitis, Pharyngitis and Adenitis (PFAPA) syndrome is the most common cause of periodic fever in childhood and was first described in 1987 by Marshall et al. [1].

This disease is characterized by periods of fever and severe inflammation, lasting for approximately 5 days, with a recurrence every three to 8 weeks [2], accompanied by aphthous stomatitis, pharyngitis, or cervical adenitis and occasional headaches, abdominal pain, and vomiting [3]. The episodes have the particular characteristic of being seemingly unprovoked, recurring periodically with an average interval between episodes of 30 days [4]. No specific diagnostic test for PFAPA is currently available and the diagnosis is based on clinical criteria [3, 5, 6]. The diagnosis is made if the patient suffers from recurring episodes of fever, accompanied by at least one of the cardinal symptoms (pharyngitis, cervical adenitis, and oral aphthosis). Episodes start before the age of 5 years and generally disappear by adulthood [7].

PFAPA shares a many clinical features with monogenic auto-inflammatory recurrent fever syndromes [8], even though no genetic causes of PFAPA have been demonstrated. PFAPA is generally considered to be a benign disease, relative to monogenetic autoinflammatory syndromes, as it has a spontaneous favorable outcome [9]. Symptomatic treatment with on-demand corticosteroids induces the rapid remission of episodes in most patients. Nonetheless, fever attacks have been shown to have a substantial impact on daily activities, schooling, and family functioning [10]. Thus, the health-related quality of life (HRQOL) is a prominent issue, even though this has not yet been studied in PFAPA patients [11].

The aim of our study was to assess the HRQOL and assess overall fatigue in children with PFAPA and compare these aspects to those of children with familial Mediterranean fever (FMF), for whom HRQOL has been shown to be worse than that of healthy controls [12–16].

## Patients and methods

### Study population

This self-administered questionnaire-based study was conducted by the National Reference Center for Auto-Inflammatory Diseases and Inflammatory Amyloidosis (CEREMAIA) at the Versailles and Kremlin Bicêtre Hospitals between November 2015 and April 2016. PFAPA patients aged between two and 18 years of age were selected from medical charts and the parents contacted by telephone and/or email. Patients were eligible for the study if: 1) they fulfilled the clinical criteria developed by Thomas & Feder et al. [1, 3, 8, 9], 2) were regularly followed, with at least one visit during the previous year, and 3) had active disease (defined by persistent recurrent fever episodes reported during the last visit). Exclusion criteria were: 1) refusal to participate, 2) a positive genetic test for autoinflammatory disease, and 3) an invalid phone and/or email address.

A group of eligible patients were identified based on the inclusion and exclusion criteria. This group was stratified by age to select aged-matched children for the control group. The control group was recruited from our cohort of pediatric FMF patients with either homozygous or compound heterozygous non-ambiguous (i.e. exon 10) *MEFV* mutations. All patients of the control group fulfilled the Tel-Hashomer criteria, were regularly on colchicine treatment, and had at least one follow-up visit during the previous year. Heterozygous patients, with a potentially less severe phenotype [17–19], were voluntarily excluded to avoid confusion between heterozygous PFAPA-like patients and our study group.

**Fig. 1:**
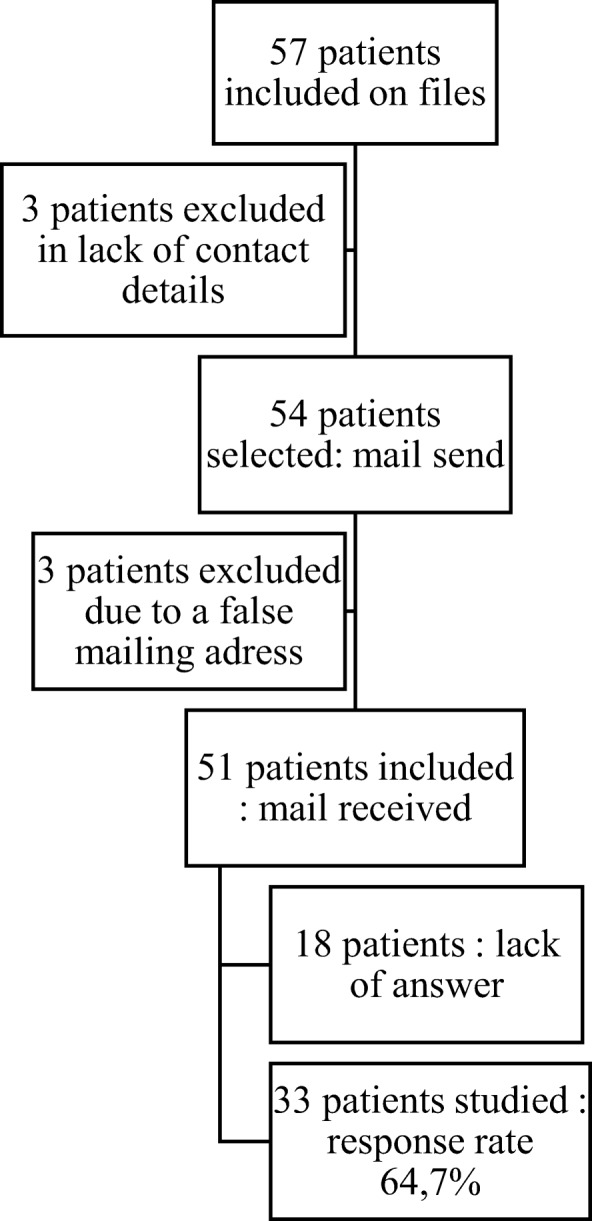
Flow chart of the recruitment of the patients

The flowchart for patient recruitment is presented in Fig. 1.

### Methods

Families of the study and control groups were contacted by email and/or telephone to recruit them to the study. An information letter was then sent by e-mail to the parents (and to the children above 8 years of age) concerning the meaning of HRQOL, the instruments, and the study protocol. Families were able to anonymously complete the parent proxy-report and the child self-reported PedsQL on a Google form with a web link included in the e-mail (Fig. 1). Once the questionnaire was completed, parents were asked to send a confirmatory email to the investigating team to avoid unnecessary reminders. The number of reminders was restricted to three per family. Neither demographic data nor clinical features were recorded, as we performed an anonymous study.

The ethics committee of the Versailles hospital (CPP) approved the study protocol.

### HRQOL assessment

We used the brief, valid and reliable multidimensional questionnaire, the Pediatric Quality of Life Inventory TM 4.0 (PedsQL™ 4.0) Generic Core Scale and the Multidimensional Fatigue Scale that use a modular approach to measure HRQOL and fatigue in children and adolescents [20–22]. These questionnaires are composed of developmentally appropriate forms for children ages 2 to 4 (assessed by a parent−/proxy-report), 5 to 7, 8 to 12, and 13 to18 years (assessed by the child and a parent/proxy report). The level of severity was calculated from the PedsQL score; higher scores indicate better HRQOL and less fatigue. The methodology has already been tested and validated by specific clinical studies performed by the MAPI research Institute, the inventor of the PedsQL [20–22]. A validated French translation of both questionnaires was used for this study.

### Statistical analysis

The data were recovered from the Google forms using Excel and then evaluated using the BiostaTGV website (http://marne.u707.jussieu.fr/biostatgv/). The data were analyzed using the Wilcoxon – Mann Whitney test. A *p*-value ≤0.05 was considered to be significant. For the sub-group analysis, patients and controls were grouped according to their ages as follows: preschool-age children (2 to 7 years) and school-age children and teens (8 to 18 years).

## Results

Thirty-three of 51 patients with PFAPA (response rate 64.7%) and 27 of 51 patients of the control group (response rate 52.9%) completed the questionnaire.

The global parent/proxy and self-reported quality of life scores were significantly lower for PFAPA patients than the control group (62.7 vs 76.4 (*p* < 0.01) see Table 1). The sub-analysis demonstrated significantly lower PedsQL™ self-reported scores of pre-school age children (2 to 7 years) with PFAPA than their FMF peers for general quality of life and physical and psychosocial functioning. The parent proxy-report did not show a significant difference, even though scores were systematically lower for PFAPA parents (Table 1). The parent proxy-report and child self-reported PedsQL™ scores of school-age children and adolescent patients (8 to 18 years) with PFAPA were lower than those of the FMF group for general quality of life and physical and psychosocial functioning; however, the difference was not significant (Table 1). The scores for the general quality of life and physical and psychosocial functioning of the child self-reported subscales correlated with those of the proxy-reported subscales (*p* = 0.81, *p* = 0.86, and *p* = 0.68, respectively for pre-school age children and *p* = 1, *p* = 0.72, and *p* = 0.69, respectively for school age children).

**Table 1:** Parent proxy-reported and child self-reported PedsQL™ scores of pediatric patients with PFAPA and FMF controls

Scale	PFAPA patients		FMF controls		*P*
	Number	Mean score	Number	Mean score	
TOTAL	33	62.7	27	76.4	< 0.01
Pre-school age children (2–7 years)					
Self-reported					
Total score	15	66.6	8	80.3	0.01
Physical health summary score	15	66.6	8	81.2	0.02
Psychosocial health summary score	15	65.6	8	80.4	0.02
Proxi-reported					
Total score	23	68.9	14	76.9	0.12
Physical health summary score	23	69.4	14	77	0.12
Psychosocial health summary score	23	68.5	14	75.5	0.27
School age children and youth(8–18 years)					
Self-reported					
Total score	8	58	13	74	0.06
Physical health summary score	8	62.5	13	72.7	0.6
Psychosocial health summary score	8	57.2	13	75.5	0.03
Proxi-reported					
Total score	10	57.4	13	72.3	0.08
Physical health summary score	10	58.8	13	71.8	0.07
Psychosocial health summary score	10	54.8	13	72.8	0.18

The scores for the general quality of life and physical and psychosocial functioning were similar between pre-school and school age children with PFAPA in the self-reported subscales (p,= 0.23, *p* = 0.65, and *p* = 0.28, respectively). For the parent proxy-reported subscales, school-age children with PFAPA had lower general quality of life and physical and psychosocial functioning scores than the younger age group (2 to 7 years) with PFAPA (p = 0.2, *p* = 0.09, and *p* = 0.16, respectively), with a significant trend.

The general fatigue scores were also found to be significantly lower for PFAPA children than their FMF peers (66.5 vs 80.3 (*p* < 0.01) see Table 2). The sub-analysis showed significantly lower self-reported fatigue scores for both pre-school and school-age children and teens with PFAPA, lower scores indicating greater fatigue.

**Table 2:** Parent proxy-reported and child self-reported fatigue scores of pediatric patients with PFAPA and FMF controls

Scale	PFAPA patients		FMF controls		*P*
	Number	Mean score	Number	Mean score	
Total	33	66.5	27	80.3	< 0.01
Pre-school age children (2/5–7 years)					
Self-reported					
Total fatigue score	15	60.4	8	78.3	0.01
General fatigue	15	70	8	84.4	0.02
Sleep/rest fatigue		73		50.5	0.02
Cognitive fatigue		81.4		61.1	0.01
Proxy-reported					
Total Fatigue score	23	69.3	14	74.9	0.27
General fatigue	23	65.8	14	73.3	0.2
Sleep/rest fatigue	23	65.9	14	74.5	0.25
Cognitive fatigue	23	76.3	14	80.8	0.33
School age children and youth (8–18 years)					
Self-reported					
Total Fatigue score	8	62	13	77.5	0.04
General fatigue	8	61.9	13	77.8	0.06
Sleep/rest fatigue	8	66.4	13	76.5	0.09
Cognitive fatigue	8	57.8	13	77.8	0.07
Proxy-reported					
Total Fatigue score	10	64.1	13	76.6	0.13
General fatigue	10	61.5	13	73.1	0.16
Sleep/rest fatigue	10	66.6	13	74.3	0.09
Cognitive fatigue	10	64.6	13	82.1	0.07

Significantly lower scores were reported in all sub-domains in preschool-age children, whereas the parent proxy-report did not find a significant difference for either age group, although the scores were systematically lower for the PFAPA group (Table 2). The total fatigue, general fatigue, sleep/rest fatigue, and cognitive fatigue scores for the child self-reported subscales correlated with those of the proxy-reported subscales (*p* = 0.96, *p* = 0.87, *p* = 0.96, and *p* = 0.53, respectively for preschool-age children and *p* = 0.83 for the total fatigue score and *p* = 0.4 for general fatigue score for school-age children and teens), but those for the sleep/rest fatigue and cognitive fatigue scores did not (*p* = 0.03 for sleep/rest fatigue and *p* = 0.01 for cognitive fatigue). The scores for total fatigue, general fatigue, sleep/rest fatigue, and cognitive fatigue were similar for both preschool and school-age children with PFAPA in the self-reported subscales (*p* = 0.76, *p* = 0.33, *p* = 0.1, and *p* = 0.72, respectively). The parent proxy-reported fatigue scores were also similar between the PFAPA school-age children and the younger age group (*p* = 0.49, *p* = 0.58, *p* = 0.84, *p* = 0.12).

## Discussion

The exact prevalence of PFAPA is not known, but the disease appears to be more common than originally thought, and PFAPA may be the most frequent recurrent autoinflammatory fever syndrome in children. Despite many patients having persistent disease for years, PFAPA is generally thought to be relatively benign. However, PFAPA patients often report non-medical problems, such as missing school due to inflammatory attacks, with a probable significant impact on the family and psychological wellbeing of the child. Despite these considerations, very few studies have focused on the quality of life of patients with recurrent autoinflammatory syndromes; even fewer for that of children and none for PFAPA children. Previously, only children with FMF were shown to have a lower than normal HRQOL [14]. Additionally, Press et al. reported that the HRQOL of parents living with a child with FMF was also less than that of parents of healthy children [11]. Thus, we compared the quality of life of children with PFAPA to that of patients with the only other autoinflammatory disease evaluated in the literature, FMF.

Our study demonstrates, for the first time that indeed, the wellbeing of PFAPA children is significantly altered, with a major negative impact on the quality of life, strongly effecting psychosocial functioning and fatigue. The patient group aged from two to 7 years reported significantly lower scores on all dimensions of the PedsQL™ (physical, emotional, social, and school functioning) than the FMF children, and the school-aged children (8 to 18 years) showed the same tendency (Table 1). Equally, the parent proxy-reported scores of all PFAPA patients (aged 2 to 7 and 8 to 18 years) were slightly lower than those reported by the parents of FMF patients, without reaching significance. Subgroup analysis showed that PFAPA patients in the 8 to 18-year-old group had significantly lower scores for psychosocial functioning, with no significant difference for physical functioning. There were no differences in either the self-reported or parent proxy-reported quality of life scores between the preschool and school-aged children.

Surprisingly, PFAPA patients showed significantly greater fatigue than their FMF peers (Table 2). Indeed, PFAPA children are considered to be completely asymptomatic between flare-ups. Low fatigue scores, corresponding to greater fatigue, could either mean that more PFAPA patients answered the questionnaire during inflammatory attacks than FMF patients or that the impact of inflammatory attacks may last beyond the child’s febrile period, even though this may have been underestimated by the caregivers. The latter hypothesis is supported by the fact that the parent proxy-reported scores did not show a significant difference in fatigue, in contrast to self-assessment. Subgroup analysis showed that PFAPA patients in the two to seven-year-old group are the most severely affected, with significantly lower scores for general, sleep/rest, and cognitive fatigue. The anonymous internet-based questionnaire design only allowed us to assume that the greater fatigue of preschool-age children was related to higher disease activity in this sub-group.

Although pediatric patient self-reporting should be considered as the standard for measuring perceived HRQOL, it is typically the parents’ perceptions of their children’s HRQOL that influences healthcare use. Several studies have reported an imperfect agreement between parent proxy and pediatric patient self-reports [23, 24]. Overall, parents consider their children to have more difficulties in physical functioning or performance, whereas there is evidence that parents may under-report their children’s difficulties in emotional functioning and fatigue. This may be explained by the fact that children’s feelings and thoughts are less noticeable than their behavior, which has more direct consequences for their family members, and that their parents are less aware of their child’s wellbeing in situations in which they are not present, such as school. However, our study showed good agreement between self-reports and proxy-reports, increasing the strength of the results.

Usually PFAPA patients are considered to be totally asymptomatic in between attacks, whereas FMF patients may have lasting clinical signs, even with regular colchicine prophylaxis. Indeed arthralgia and/or myalgia, lasting a few hours to an entire day, is frequent for pediatric FMF patients following a physical exertion or prolonged standing [25]. Former studies have shown that such musculoskeletal manifestations affect the HRQOL scores of FMF children [14]. It is thus highly significant that the reported HRQOL of PFAPA patients in our study is even lower than that of FMF patients.

The main limitation of our study was its anonymous questionnaire-based design. An anonymous questionnaire made it possible to evaluate the patients in their daily living conditions, but it deprived us of performing a finer analysis of the results by combining them with activity and severity markers. Nonetheless, our study only intended to provide a first glimpse of the quality of life of PFAPA patients and demonstrates, for the first time, that the impact of the disease is most likely underestimated. Physicians should strive to consider the fact that PFAPA is an intermittent disease without preventive treatment options. Although the quality of life scores may be dependent on whether they were collected during an inflammatory or asymptomatic period, the persistence of regular inflammatory attacks is the main reason for the lower quality of life scores. It is indeed likely that our control group (FMF patients on regular colchicine treatment) experienced less inflammatory attacks during the study period than the PFAPA patients, explaining the lower impact of the disease on general wellbeing.

Further limitations of our study include the small sample size and some difficulties to include patients in our control group. Indeed, FMF patients were less prone to respond to our survey, with a significantly lower response rate (52.9% vs 64.7% for PFAPA patients). It is possible that FMF patients were less motivated to answer our survey, knowing that they were the control group. However, they may also have considered that HRQOL is not a problem in FMF. If so, this may have introduced a bias to our results, as severe patients may have been more likely to respond to our survey. In this case, our results are even more striking, showing a greater impairment of the quality of life of PFAPA patients. Our study design did not allow us to correlate HRQOL scores to validated severity or activity scores, preventing us from assessing this hypothesis.

## Conclusion

The results of this pilot study to assess the HRQOL of PFAPA children and adolescents refutes, for the first time, the belief that PFAPA is a benign disease and demonstrates that the seriousness of PFAPA has probably been largely underestimated. Indeed, the HQROL scores in our study were lower than those of patients with FMF, a chronic genetic lifelong auto-inflammatory fever syndrome. This finding has potential clinical significance for the healthcare needs of children and adolescents with PFAPA. Special and individual counselling and intervention is needed, given the degree of the reported impairment of their HRQOL. Further multicentric studies are necessary to validate our results and correlate the quality of life to disease activity scores.

## Abbreviations

CEREMAIA: National Reference Center for Auto-Inflammatory Diseases and Inflammatory Amyloidosis
FMF: familial Mediterranean fever
HRQOL: health-related quality of life
PedsQL™ 4.0: Pediatric Quality of Life Inventory TM 4.0
PFAPA: periodic fever, aphthous stomatitis, pharyngitis, and adenitis
